# Investigation of Anomalous Degradation Tendency of Low-Frequency Noise in Irradiated SOI-NMOSFETs

**DOI:** 10.3390/mi14030602

**Published:** 2023-03-04

**Authors:** Rui Liu, Linchun Gao, Juanjuan Wang, Tao Ni, Yifan Li, Runjian Wang, Duoli Li, Jianhui Bu, Chuanbin Zeng, Bo Li, Jiajun Luo

**Affiliations:** 1 Institute of Microelectronics, Chinese Academy of Sciences, Beijing 100029, China; 2 University of Chinese Academy of Sciences, Beijing 100049, China; 3 Key Laboratory of Science and Technology on Silicon Devices, Chinese Academy of Sciences, Beijing 100029, China

**Keywords:** low-frequency noise, ionizing radiation, radiation effects, partially depleted silicon-on-insulator (PDSOI), MOSFET, border trap, interface trap

## Abstract

In this work, we present new evidence of the physical mechanism behind the generation of low-frequency noise with high interface-trap density by measuring the low-frequency noise magnitudes of partially depleted (PD) silicon-on-insulator (SOI) NMOSFETs as a function of irradiation dose. We measure the DC electrical characteristics of the devices at different irradiation doses and separate the threshold-voltage shifts caused by the oxide-trap charge and interface-trap charge. Moreover, the increased densities of the oxide-trap charge projected to the Si/SiO_2_ interface and interface-trap charge are calculated. The results of our experiment suggest that the magnitudes of low-frequency noise do not necessarily increase with the increase in border-trap density. A novel physical explanation for the low-frequency noise in SOI-NMOSFETs with high interface-trap density is proposed. We reveal that the presence of high-density interface traps after irradiation has a repressing effect on the generation of low-frequency noise. Furthermore, the exchange of some carriers between border traps and interface traps can cause a decrease in the magnitude of low-frequency noise when the interface-trap density is high.

## 1. Introduction

Recently, silicon-on-insulator (SOI) technology has become increasingly important in radiation-exposed environments such as space and military electronics due to its greater immunity to single-event effect (SEE) and suppression of the latch-up effect [1,2,3]. Furthermore, due to their strong anti-interference properties, high integration density, and fast operation speed, SOI MOSFETs have been found to be applicable to signal processing circuits, especially in radiation-exposed settings [4,5]. It is well known that low-frequency noise plays a substantial role in the performance of low-noise signal processing circuits [6]. However, as devices become increasingly scaled down, noise emerges as a crucial factor affecting device and circuit performance. Thus, exploring the electrical characteristics and low-frequency noise of SOI MOSFETs under irradiation, in addition to the underlying physical mechanisms, is of critical importance. As devices are miniaturized, it has been observed that thinner gate oxide becomes less susceptible to the total ionizing dose (TID) effect, leading to decreased threshold-voltage shifts and leakage current [7]. Despite this, TID still affects the low-frequency noise of smaller devices, related to interface traps at the Si/SiO₂ interface and border traps in oxide near the interface (2–3 nm). Some studies have focused on the low-frequency noise of MOSFETs under irradiation [7,8,9,10,11,12,13,14,15]. However, most of these studies only compared the low-frequency noise of devices before and after irradiation with a certain dose, and few have studied the degradation trend of the low-frequency noise of devices in a wider range of radiation doses.

Two mechanisms are mainly responsible for the low-frequency noise in semiconductor devices: the mobility fluctuation model and the carrier number fluctuation model. The low-frequency noise due to fluctuation of carrier mobility in the channel caused by various scattering processes, referred to as fundamental 1/f noise, and the low-frequency noise due to fluctuation of the carrier number caused by defects traps, referred to as nonfundamental 1/f noise, are observed in MOSFETs. Nonfundamental 1/f noise usually has a much larger magnitude than fundamental 1/f noise. Moreover, fundamental 1/f noise dominates only when the defect density is reduced enough to ignore nonfundamental 1/f noise [16]. Therefore, it is generally assumed that the low-frequency noise in MOSFETs is caused by the fluctuation of carrier number. The theoretical sources of the fluctuation in carrier number mainly comprise the following [17]. McWhorter suggested that tunneling exchange of carriers between the channel and border traps of the same energy level is the primary generator of low-frequency noise [18]. Sah et al. then proposed an alternative tunneling model, which utilized interface traps as intermediate states during the tunneling process [19]. Apart from the tunneling model, Dutta and Horn determined that low-frequency noise also could be attributed to the trapping of carriers thermally activated by border traps [20]. However, tunneling and Dutta-Horn models both fail to explain low-frequency noise of a clean silicon surface in a vacuum environment [21]. To explain the low-frequency noise generated without an oxide layer or suitable traps inside the oxide layer, Jäntsch suggested that low-frequency noise is generated through the random walk of electrons at the interface [22]. As mentioned above, both border traps and interface traps contribute to low-frequency noise in MOSFETs. Moreover, the tunneling theory remains widely accepted.

The work of Sah et al. demonstrates that carriers liberated by interface traps can be caught by border traps [19]. Additionally, the random walk theory exhibits that the time constants of interface traps can be modulated to be close to that of border traps by carriers randomly walking at the interface [22]. These previous investigations have given the physical basis of carriers exchanging between interface traps and border traps. However, few research addresses the effect of increasing interface traps on the low-frequency noise of MOSFETs after irradiation.

In this paper, the measurements of low-frequency noise behavior and DC electrical characteristics of SOI NMOSFETs of different sizes and gate oxide thicknesses at irradiation doses are presented, serving as a valuable reference for circuit design applications that necessitate good noise performance in radiation conditions. Furthermore, analysis of the low-frequency noise magnitudes post-irradiation is conducted. Additionally, a novel physical mechanism is suggested for low-frequency noise in NMOSFETs with a high density of interface traps.

## 2. Devices and Experimental Details

### 2.1. Devices under Test

The devices we tested were SOI-NMOSFETs with an operating voltage of 1.8 V and 5 V, which were fabricated using a 0.18-μm PD-SOI CMOS process. The gate lengths *L* = 0.18 μm and 10 μm for 1.8 V devices and *L* = 0.5 μm and 10 μm for 5 V devices. Table 1 outlines the main parameters of the device under test.

**Figure 1:**
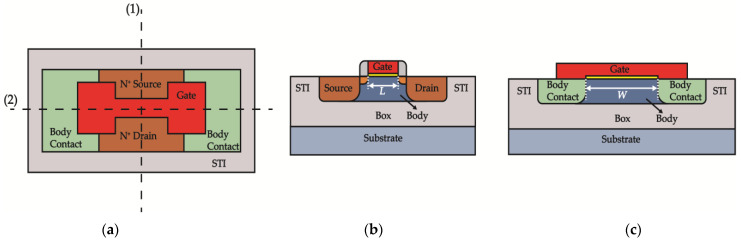
(**a**) Layout of measured devices. (**b**) Cross-sectional view of measured devices along the dashed line (1) in (**a**). (**c**) Cross-sectional view of measured devices along the dashed line (2) in (**a**).

The layout and its corresponding cross-sectional view of these SOI NMOSFETs are illustrated in Figure 1. The layout of the measured devices employs an edgeless H-gate structure, hence no overlap between the gate and the shallow trench isolation (STI) is present, thereby eliminating the influence STI may have on the channel after irradiation. Additionally, each device has a body contact to eliminate the floating-body effect.

### 2.2. Experimental Details

**Figure 2:**
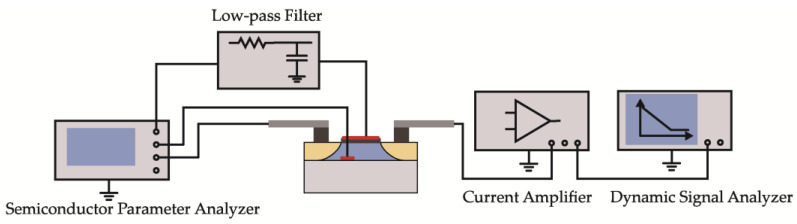
Illustration of the low-frequency noise measurement system.

To minimize experimental error and increase the reliability of the results, we tested three devices of each size in the following experiments. All devices were irradiated to 1 Mrad (Si) of γ-rays from a ^60^Co source at a dose rate of 100 rad (Si)/s and a temperature of ~298 K. During irradiation, all devices were biased in an ON-state, with the gate held at *V*_DD_ and the other terminals grounded. The electrical and noise performance measurements were conducted within two hours after each exposure step. The electrical characterization was measured using the Keithley 4200-SCS Semiconductor Parameter Analyzer at room temperature. The threshold voltage (*V*_th_) was obtained via linear extrapolation (LE) [23], whereby the *V*_g_ axis intercept of the linear extrapolation of the *I*_d_-*V*_gs_ curve at the point with the largest first derivative was calculated when the device was in the linear region (*V*_ds_ = 100 mV), such that *V*_th_ equaled the intercept minus *V*_ds_/2. Moreover, the threshold-voltage shift (∆*V*_th_) of the device after irradiation was partitioned into the components due to trapped charge in oxide traps (∆*V*_ot_) and interface traps (∆*V*_it_), per the midgap charge method presented by McWhorter [24]. Additionally, the drain-current noise power density spectral *S*_id_ of the devices was measured with a noise measurement system at room temperature. The drain-current noise signal was amplified by the low-noise current amplifier SR570 and then measured by the HP35670 dynamic signal analyzer. Figure 2 illustrates the schematic diagram of the noise measurement system used in this experiment.

## 3. Results and Discussion

**Figure 3:**
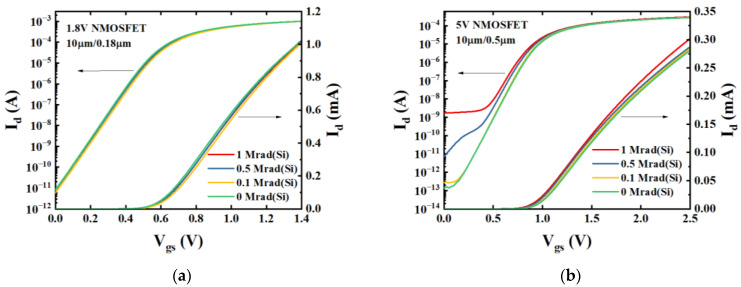
*I*_d_–*V*_gs_ curves in the linear region (*V*_ds_ = 0.1 V) of (**a**) 1.8 V NMOSFETs for geometry: *W*/*L* = 10 μm/0.18 μm and (**b**) 5 V NMOSFETs for geometry: *W*/*L* = 10 μm/0.5 μm. All devices were irradiated up to 1 Mrad(Si) under the “on” bias condition.

Figure 3 displays the linear and logarithmic coordinates of the *I*_d_-*V*_gs_ electrical characteristics of NMOSFETs in the linear region (*V*_ds_ = 0.1 V) for operating voltages of 1.8 V and 5 V. The *I*_d_-*V*_gs_ curves of 1.8 V NMOSFETs with H-gate have been observed to remain insensitive to increasing radiation dose up to 1 Mrad. In contrast, the off-state current of 5 V NMOSFETs with H-gate has been found to vary more significantly in response to a radiation dose of up to 1 Mrad.

**Figure 4:**
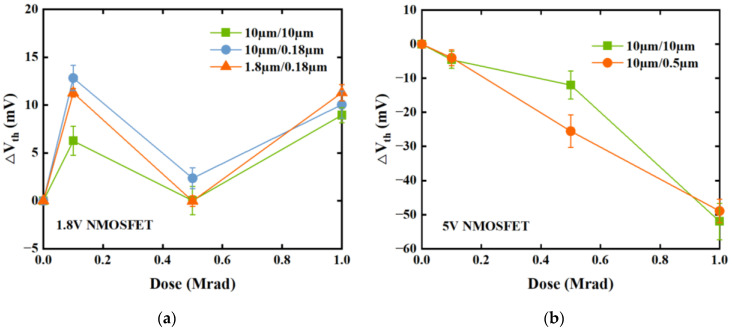
Threshold-voltage shift ∆*V*_th_ as a function of irradiation dose for (**a**) 1.8 V NMOSFETs with geometries *W*/*L* = 10 μm/10 μm, 10 μm/0.18 μm, and 1.8 μm/0.18 μm and (**b**) 5 V NMOSFETs with geometries *W*/*L* = 10 μm/10 μm and 10 μm/0.5 μm. All devices were irradiated up to 1 Mrad(Si) under the “on” bias condition.

The *V*_th_ of all the devices tested are calculated using linear extrapolation (LE) [23]. Figure 4 shows the ∆*V*_th_ of 1.8 V and 5 V devices of different sizes as a function of irradiation dose. Figure 4a demonstrates that the ∆*V*_th_ of each 1.8 V device rises from pre-irradiation up to 0.1 Mrad, then falls from 0.1 Mrad to 0.5 Mrad before increasing once more from 0.5 Mrad to 1 Mrad. Figure 4b reveals that the ∆*V*_th_ of each 5 V device decreases with the increase in doses. Overall, it was determined that the ∆*V*_th_ of 1.8 V devices are comparatively less significant than those of 5 V devices.

**Figure 5:**
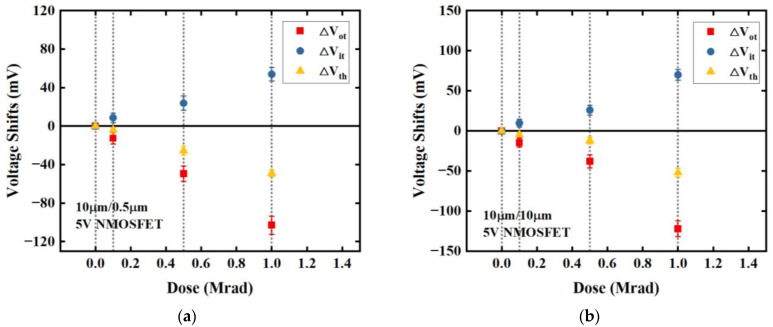
Net threshold-voltage shifts and components due to oxide-trap charge and interface-trap charge as a function of irradiation dose for the 5 V NMOSFETs (**a**) with a width of 10 μm and a length of 0.5 μm, as well as those (**b**) with a width of 10 μm and a length of 10 μm. All devices were irradiated up to 1 Mrad(Si) under the “on” bias condition.

∆*V*_th_ is contributed by both the interface-trap and oxide-trap charge, as demonstrated by the equation below.:

(1) $$\begin{array}{c} {\Delta V}_{\mathrm{th}}={\Delta V}_{\mathrm{it}\;}{+\Delta V}_{\mathrm{ot}\;} \end{array}$$

where ∆*V*_it_ is the threshold-voltage shift due to net interface-trap charge and ∆*V*_ot_ is the threshold-voltage shift due to net oxide-trap charge. We split the threshold-voltage shift into ∆*V*_it_ and ∆*V*_ot_ using the midgap charge method [23]. However, the midgap voltage of the 1.8 V device is barely detectable due to the trivial variation between the subthreshold-current curves of different irradiation doses. Figure 5 depicts the threshold-voltage shifts caused by net oxide-trap and interface-trap charge as a function of irradiation dose for 5 V NMOSFETs with geometries of (a) *W*/*L* = 10 μm/0.5 μm and (b) 10 μm/10 μm.

Once the ∆*V*_it_ due to the net interface-trap charge has been obtained, the increased density of interface-trap charge can be calculated using the following equation:

(2) $$\begin{array}{c} {\Delta N}_{\mathrm{it}}=\frac{{\Delta V}_{\mathrm{it}}C_{\mathrm{ox}}}{q} \end{array}$$

where *C*_ox_ is the capacitance per unit area of the gate oxide, and *q* is the electron charge. Similarly, once the ∆*V*_ot_ due to the net oxide-trap charge is obtained, the increased density of the oxide-trap charge projected to the Si/SiO_2_ interface can be calculated using the following equation:

(3) $$\begin{array}{c} {\Delta N}_{\mathrm{ot}}=\frac{{\Delta V}_{\mathrm{ot}}C_{\mathrm{ox}}}{q} \end{array}$$

Although the ∆*V*_th_ of a 5 V device became increasingly negative with an increasing irradiation dose, the ∆*V*_it_ due to interface traps became correspondingly more positive. This implies that the densities of both interface traps and oxide traps increase with the rising irradiation dose. Table 2 displays the corresponding values of ∆*N*_it_ and ∆*N*_ot_ calculated from the ∆*V*_it_ and ∆*V*_ot_ in Figure 5.

**Figure 6:**
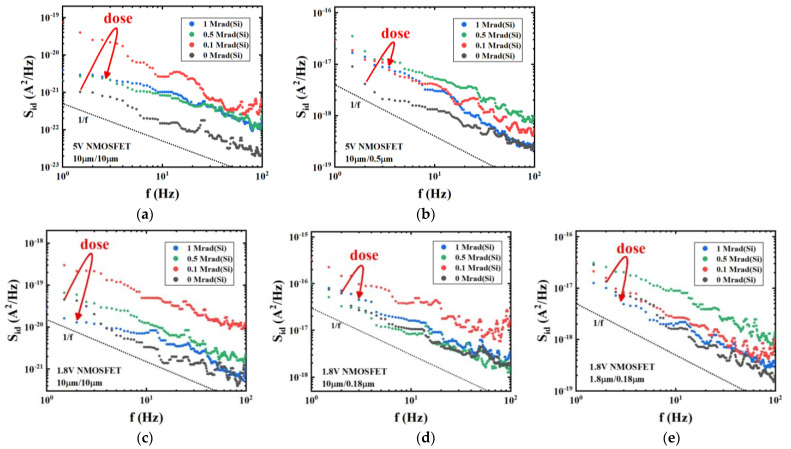
Drain-current noise power density spectrum of 5 V NMOSFETs for geometries: (**a**) *W*/*L* = 10 μm/10 μm and (**b**) *W*/*L* = 10 μm/0.5 μm and 1.8 V NMOSFETs for geometries: (**c**) *W*/*L* = 10 μm/10 μm, (**d**) *W*/*L* = 10 μm/0.18 μm and (**e**) *W*/*L* = 1.8 μm/0.18 μm at different irradiation doses. All devices were irradiated up to 1 Mrad(Si) under the “on” bias condition. Noise measurements were carried out when *V*_gs_ − *V*_th_ = 0.3V and *V*_ds_ = 0.1V.

Figure 6 shows the drain-current noise power density spectrum of 1.8 V and 5 V NMOSFETs with different dimensions before and after irradiation with different doses. We measured the low-frequency noise between 1 Hz and 100 Hz in the linear region of device response where drain voltage *V*_ds_ = 0.1 V and gate voltage *V*_gt_ = *V*_gs_ − *V*_th_ = 0.3 V. The low-frequency noise of all measured devices is characterized by 1/*f* noise. By comparing the pre-irradiation drain-current low-frequency noise magnitudes of the devices in Figure 6a,b, and those in Figure 6c,d, it can be seen that the device with the shorter gate length exhibits a higher low-frequency noise at the same gate width. Similarly, comparison of the pre-irradiation drain-current low-frequency noise magnitudes of the devices in Figure 6d,e reveals that the device with the wider gate width exhibits a higher low-frequency noise at the same gate length. These correlations observed between the device dimensions and the low-frequency noise are consistent with the previous physical explanation that low-frequency noise in MOSFETs originates from the exchange of carriers between the channel and border traps [18,25,26]. The trend of low-frequency noise as a function of the irradiation dose is largely similar for all measured devices in Figure 6. The noise magnitudes of all devices reach a peak at a comparatively low irradiation dose, which is 0.5 Mrad for the 5 V device with geometry *W*/*L* = 10 μm /0.5 μm and 1.8 V device with geometry *W*/*L* = 1.8 μm /0.18 μm, and 0.1 Mrad for the other devices. Furthermore, the noise magnitudes at higher irradiation doses are generally lower than those at lower doses, even though the density of border traps in the oxide tends to increase at higher radiation doses. Interestingly, this trend appears to contradict the previously proposed physical mechanism of low-frequency noise that carriers exchange between the channel and border traps [18,19,20].

The tunneling model for low-frequency noise can be expressed by a first-order expression, as outlined by its physical mechanism, which indicates that carriers transition between the channel and border traps. This is expressed as follows: [27,28]:

(4) $$\begin{array}{c} S_{\mathrm{vd}}=\frac{q^{2}}{C_{\mathrm{ox}}^{2}}\frac{V_{D}^{2}}{{\left(V_{G}{-V}_{\mathrm{th}}\right)}^{2}}\frac{k_{B}{TD}_{\mathrm{bt}}\left(E_{f}\right)}{LW\ln\left(\tau_{1}/\tau_{0}\right)}\frac{1}{f} \end{array}$$

where *S*_vd_ is drain-voltage noise power density spectrum, *k*_B_ is Boltzmann’s constant, *T* is absolute temperature, *D*_bt_(*E*_f_) is the density of border traps in oxide near the interface (2–3 nm) at Fermi level, *f* is frequency, and *τ*_0_ and *τ*_1_ are minimum and maximum tunneling time respectively. In the linear region of a device, the relationship between drain-voltage noise power density spectrum *S*_vd_ and drain-current noise power density spectrum *S*_id_ is given by:

(5) $$\begin{array}{c} S_{\mathrm{id}}=\frac{I_{\mathrm{ds}}^{2}}{V_{\mathrm{ds}}^{2}}S_{\mathrm{vd}} \end{array}$$

where *I*_ds_ is drain-current, and *V*_ds_ is drain-voltage.

**Figure 7:**
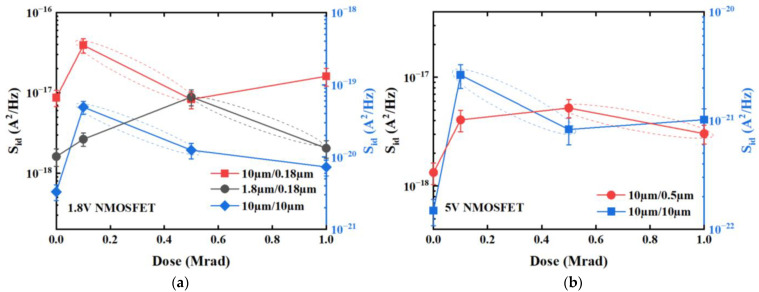
Drain-current noise magnitudes at *f* = 10 Hz as a function of radiation dose. (**a**) 1.8 V NMOSFETs for geometries: *W*/*L* = 10 μm/10 μm, 10 μm/0.18 μm and 1.8 μm/0.18 μm, and (**b**) 5 V NMOSFETs for geometries: *W*/*L* = 10 μm/10 μm and 10 μm/0.5 μm. All devices were irradiated up to 1 Mrad(Si) in the “on” bias condition. Noise measurements were carried out when *V*_gs_ − *V*_th_ = 0.3 V and *V*_ds_ = 0.1 V.

According to the first-order expression of the tunneling model, as the border traps increase with increasing irradiation dose, the *S*_id_ should have increased accordingly. However, Figure 6 appears to contradict this prediction, as noise magnitudes at higher irradiation doses decrease. This phenomenon is further demonstrated in Figure 7 which shows that drain-current noise magnitudes of 1.8 V and 5 V NMOSFETs at a frequency of 10 Hz reach a maximum at a certain irradiation dose before decreasing with further increases in radiation exposure. Figure 7 shows that the magnitude of the 10 Hz drain-current noise of a 1.8 V NMOSFET with a size of *W*/*L* = 10 μm/0.18 μm reaches its maximum value 3.91 × 10^−17^ A^2^/Hz at 0.1 Mrad, then decreases to 8.30 × 10^−18^ A^2^/Hz at 0.5 Mrad; for a 1.8 V NMOSFET with a size of *W*/*L* = 10 μm/10 μm, the maximum value is 4.91 × 10^−20^ A^2^/Hz at 0.1 Mrad and decreases to 1.25 × 10^−20^ A^2^/Hz at 0.5 Mrad; for a 1.8 V NMOSFET with a size of *W*/*L* = 1.8 μm/0.18 μm, the maximum value is 8.88 × 10^−18^ A^2^/Hz at 0.5 Mrad and decreases to 2.04 × 10^−18^ A^2^/Hz at 1 Mrad; for a 5 V NMOSFET with a size of *W*/*L* = 10 μm /10 μm, the maximum value is 2.62 × 10 ^−21^A^2^/Hz at 0.1Mrad and decreases to 8.28 × 10 ^−22^A^2^/Hz at 0.5 Mrad; finally, for a 5 V NMOSFET with a size of *W*/*L* = 10 μm /0.5 μm, the maximum value is 5.22 × 10 ^−18^A^2^/Hz at 0.5Mrad dose and decreases to 3.03 × 10 ^−18^ A^2^/Hz at 1 Mrad. Therefore, it appears that factors beyond those accounted for by carriers exchanging between border traps and the channel may be influencing the noise magnitude. We shall further analyze the generation mechanism of low-frequency noise at higher interface-trap densities.

The origin of low-frequency noise has been attributed to the number fluctuations of carriers in the channel [18,29]. However, there are various theories regarding the physical mechanism that is responsible for this fluctuation. One theory suggests that carriers are temporarily captured by border traps located in the oxide near the Si/SiO_2_ interface (2–3 nm) before they are released back into the channel, which is widely accepted [26,30,31]. According to this theory, a positively charged border trap after irradiation is believed to be a stable state that can exchange a carrier with the channel [32,33]. However, this theory does not take into account the effect of interface traps on the exchange of carriers between border traps and the channel. Another theory proposes that carriers are captured by interface traps with a short time constant and emitted into their vicinity where they randomly wander for an extended period until they are recaptured by an interface trap and eventually released back into the channel [22]. However, this random walk concept only considers cases where there is no presence of an oxide layer or suitable traps in oxide layer, disregarding any effect from border traps.

In our experiments, the magnitude of the low-frequency noise after irradiation is indeed higher than before irradiation, which can indicate that the low-frequency noise is related to the increased border traps in the oxide layer [18,19,20]. However, the experimental phenomenon that the magnitudes of low-frequency noise at high irradiation doses are lower than those at low irradiation doses cannot be explained by border traps in the oxide layer alone. Table 2 shows that as the irradiation dose increases, the number of both oxide traps and interface traps increase. Combining with the previous theoretical basis [19,22], we propose a new physical mechanism of low-frequency noise at high interface-trap density, which combines the roles of interface traps and border traps.

**Figure 8:**
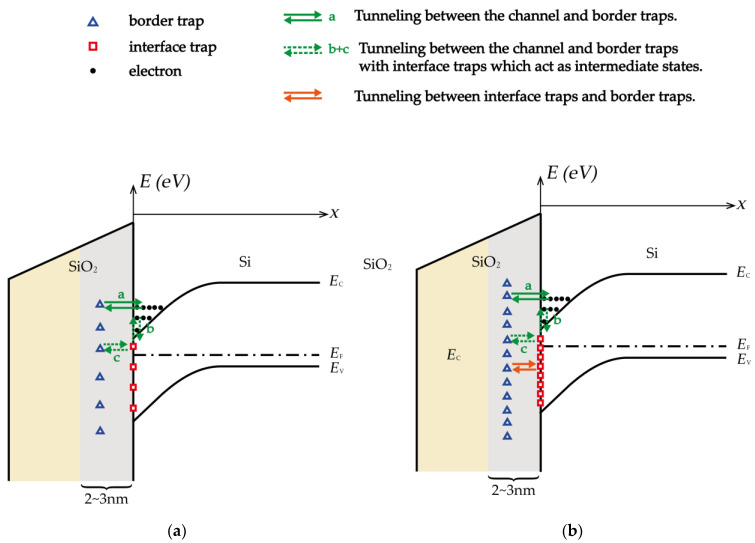
The physical processes of the origin of low-frequency noise in NMOSFETs under (**a**) pre-irradiation/lower-dose irradiation and (**b**) higher-dose irradiation in the energy band diagram.

Figure 8 shows the physical processes of the origin of low-frequency noise in NMOSFETs when exposed to pre-irradiation/lower-dose irradiation and higher-dose irradiation, as depicted in the energy band diagram. Figure 8a illustrates the mechanism of low-frequency noise generation under pre-irradiation/low-dose irradiation, wherein the interface-trap density is relatively low, and the source of low-frequency noise mainly originates from carrier exchange between the channel and border traps, resulting in fluctuation of carrier number in the channel. Consequently, at lower doses of irradiation, the factor that leads to an increase in the magnitude of low-frequency noise relative to before irradiation is mainly due to increased border traps. Figure 8b shows the mechanism of low-frequency noise generation at higher doses of irradiation, where both interface traps and border traps increase with increasing doses, which makes it more likely for carriers to exchange between interface traps and border traps. This kind of carrier exchange between interface trap and border trap is equivalent to reducing the number of border trap that should have exchanged carriers with the channel. Therefore, at higher doses of irradiation, increased interface trap has a certain degree of inhibitory effect on the low frequency noise generated by border trap.

**Figure 9:**
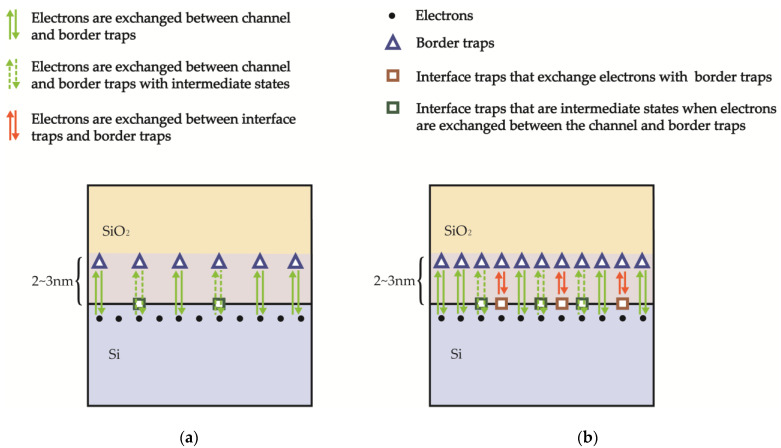
Illustration of low-frequency noise generation in NMOSFETs (**a**) before/under lower-dose irradiation, (**b**) under higher-dose irradiation.

Figure 9 illustrates the schematic of low-frequency noise generation in nMOSFETs. Figure 9a displays the situation where interface traps have not yet inhibited the exchange of electrons between the channel and border traps, with the border-trap density alone resulting in the generation of low-frequency noise before/under lower-dose irradiation. Figure 9b depicts the situation under higher-dose irradiation, where interface traps have already hindered electron exchange between the channel and border traps, such that the density of border traps which actually contribute to generation of low-frequency noise is equal to the total density of border traps minus the part which undergoes electron exchange with interface traps, as shown in Equation (6):

(6) $$\begin{array}{c} N_{\mathrm{bt}}^{\prime }=N_{\mathrm{bt}}-P\cdot N_{\mathrm{it}} \end{array}$$

where $N_{\mathrm{bt}}^{\prime }$ is the density of border traps which actually contribute to generation of low-frequency noise, *N*_bt_ is the total border-trap density, *N*_it_ is the interface-trap density, and *P* is the proportion of interface traps that exchange electrons with border traps to the total interface traps.

Therefore, based on the physical mechanism of low-frequency noise generation described above in the case of high interface-trap density, the first-order tunneling model of low-frequency noise at high interface-trap density should be modified accordingly. The *D*_bt_(*E*_f_) in Equation (4) should be replaced with the *D*_bt_′(*E*_f_) which is the density of border traps that truly contribute to low-frequency noise at Fermi level, as shown in the following equation:

(7) $$\begin{array}{c} D_{\mathrm{bt}}^{\prime }\left(E_{f}\right)\approx \frac{N_{\mathrm{bt}}^{\prime }}{E_{g}} \end{array}$$

where *N*_bt_’ is the density of border traps that truly contribute to low-frequency noise, as shown in Equation (6), and *E*_g_ is the energy band gap width. Then the expression of first-order tunneling model should accordingly be modified to the following expression:

(8) $$\begin{array}{c} S_{\mathrm{vd}}=\frac{q^{2}}{C_{\mathrm{ox}}^{2}}\frac{V_{D}^{2}}{{\left(V_{G}{-V}_{\mathrm{th}}\right)}^{2}}\frac{k_{B}{TD}_{\mathrm{bt}}^{\prime }\left(E_{f}\right)}{LW\ln\left(\tau_{1}/\tau_{0}\right)}\frac{1}{f} \end{array}$$

Equations (6)–(8) indicate that two additional parameters, *P* and *N*_bt_, are required to quantify the magnitude of low-frequency noise. It has been observed that the trend of ∆*N*_bt_ and ∆*N*_ot_ both increase with increasing irradiation dose, implying that the ratio of ∆*N*_bt_ to ∆*N*_ot_ is a constant value [34]. Here, we use the ∆*N*_bt_/∆*N*_bt_ of 18% to characterize the low-frequency noise magnitude of 5 V devices, which is consistent with previous measurements [34]. Additionally, as irradiation dose increases, *P*, the proportion of interface traps that exchange electrons with border traps to the total interface traps also increases. The *P* and ∆*N*_bt_/∆*N*_ot_ used for validating our low-frequency noise mechanism are provided in Table 3.

**Figure 10:**
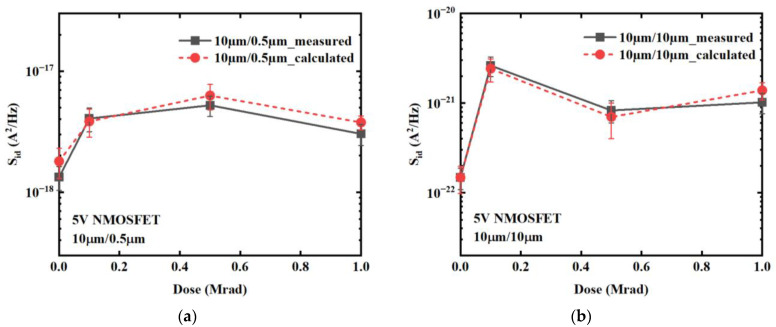
Noise magnitudes at *f* = 10 Hz of 5 V NMOSFETs for geometries: (**a**) *W*/*L* = 10 μm/0.5 μm and (**b**) 10 μm/10 μm as a function of irradiation dose which are measured and calculated under the modified physical mechanism.

The border-trap density, *N*_bt_, can be expressed as *N*_bt_ = *N*_bt0_ + ∆*N*_bt_, where *N*_bt0_ ≈ *N*_bt0′_ is the initial density of border traps before irradiation and can be calculated using Equations (7) and (8) with pre-irradiation noise magnitude and an assumed *τ*_1_/*τ*_0_ ratio of ~10^12^ [9]. For 10 μm/10 μm and 10 μm/0.5 μm devices, the values of *N*_bt0_ are 2.40 × 10^8^ cm^−2^ and 1.71 × 10^9^ cm^−2^ respectively. The interface-trap density is rough ~10^9^ cm^−2^ prior to irradiation, which is an order of magnitude smaller than after irradiation, thus *N*_it_ ≈ ∆*N*_it_ post irradiation. By utilizing Equations (5)–(8), the low-frequency noise magnitude as a function of dose was calculated using the low-frequency noise magnitude before irradiation, data from Table 2, and parameters from Table 3. Figure 10 presents the magnitude of noise at 10 Hz for 5 V NMOSFETs as a function of radiation dose, which were both measured and calculated under the physical mechanism established in this study. Figure 10 demonstrates that the trend of low-frequency noise magnitudes calculated through this physical mechanism is consistent with that observed in the measured values, thus confirming the correctness and efficacy of this proposed physical mechanism.

Therefore, this new physical mechanism can explain the low-frequency noise magnitude as a function of irradiation dose in our experiment. Before irradiation and at a dose of 0.1 Mrad, the low-density interface traps had little effect on the exchange of carriers between the channel and border traps, thus making the main source of impact on low-frequency noise still that of increased border traps. As the dose increased, however, this exchange was partially suppressed by the increased interface traps, resulting in lower magnitudes of low-frequency noise at higher doses compared to those seen at lower doses. Furthermore, for certain devices, the dose at which their noise magnitude attained its maximum is greater than that observed for other devices. This is due to the fact that, despite some carriers exchanging between border traps and interface traps at this dose, the density of border traps that truly contributed to carrier number fluctuation in the channel at this dose is higher than that at lower doses. The low-frequency noise magnitudes of some devices increase at 1 Mrad for the same reason.

## 4. Conclusions

We have conducted a comprehensive investigation into the low-frequency noise of SOI-NMOSFETs across varied irradiation doses. The measurements indicate that the low-frequency noise magnitudes of all devices tend to see an increase, then decrease with increasing irradiation dose, suggesting that the density of border traps is no longer the only deciding factor in the low-frequency noise magnitude at higher irradiation doses. We point out that the enhanced interface traps cause more carriers to exchange between the interface traps and border traps, thereby suppressing the exchange of carriers between the channel and border traps, which results in the lower magnitude of low-frequency noise at higher irradiation doses. This research provides an important understanding of the physical mechanism of low-frequency noise with high interface-trap density, as well as relevant guidance for low-noise circuit designs in irradiated environments.

## Figures and Tables

**Table 1:** Main parameters of the SOI-NMOSFETS under test.

Parameter	Value of 1.8 V Devices	Value of 5 V Devices
Operating Voltage	1.8 V	5 V
Gate Length (*L*)	0.18 μm, 10 μm	0.5 μm, 10 μm
Gate Width (*W*)	1.8 μm, 10 μm	10 μm
Gate Oxide thickness (*T*_ox_)	4 nm	12.5 nm
Channel Doping (*N*_channel_)	1 × 10^18^ cm^−3^	2 × 10^17^ cm^−3^

**Table 2:** ∆*N*_it_ and ∆*N*_ot_ of devices in Figure 5.

*W/L*	Dose (Mrad)	∆*N*_it_ (10^10^ cm^−2^)	∆*N*_ot_ (10^10^ cm^−2^)
10 μm/10 μm	0.1	1.72	2.52
	0.5	4.83	6.58
	1	12	21.4
10 μm/0.5 μm	0.1	1.52	2.22
	0.5	4.18	8.65
	1	9.44	18

**Table 3:** ∆*N*_bt_/∆*N*_ot_ of the 5 V NMOSFETs under test and proportion of interface traps that exchange carriers with border traps to the total interface traps.

Dose (Mrad)	∆*N*_bt_/∆*N*_ot_	*P*
0.1	18%	5%
0.5	18%	22%
1	18%	31%
